# Misadventure during laparoscopic sleeve gastrectomy: why it happened? how to prevent and recover from it?

**DOI:** 10.1590/0102-6720201600S10032

**Published:** 2016

**Authors:** Deborshi SHARMA, Priya HAZRAH, Swati SATTAVAN, Pavitra Kumar GANGULY, Romesh LAL

**Affiliations:** 1 Department of Surgery, Lady Hardinge Medical College & Associated Dr RML Hospital India; 2 Department of Surgery, Hamdard Institute of Medical Sciences and Research, New Delhi, India

**Keywords:** Laparoscopic sleeve gastrectomy, Complications, Nasogastric tube

## INTRODUCTION

Laparoscopic sleeve gastrectomy (LSG) is gaining popularity as a single stage procedure for morbid obesity^1^. It is regarded as a safer procedure than other more complex procedures as it avoids the long-term micronutrient deficiency^1^. LSG can present with major complications in up to 29% and among them, the staple line leak can be in 0-7%^2.^ Here is reported a very rare and unusual complication with LSG which is completely preventable. 

## CASE REPORT

**FIGURE 1:**
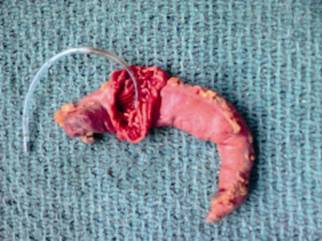
Resected sleeve specimen showing the nasogastric tube embedded in the stapler line

A middle aged women with history of old inferior wall myocardial infarction and BMI 42 underwent LSG. She had an eventless intraoperative period and was used five fires of green and blue staplers to cover the length of the sleeve. After, checking for any leak using the air leak test was done. At that time a moderate alarm was sounded by the peri-operative physicians that the nasogastric (NG) tube was struck somewhere as it was not coming out. Immediately by focusing on the remnant gastric sleeve a dimple was noticed around the mid of the sleeve every time the anaesthetist was trying to pull the tube out. The extracted gastric greater specimen was examined by opening the greater curve to find the severed distal end of the NG tube firmly stapled (Figure 1). 

**FIGURE 2:**
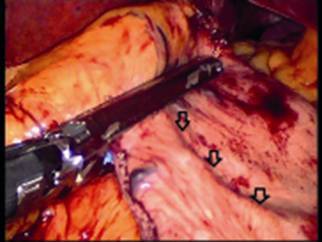
Retrospectively analyzed video image showing the nasogastric tube (arrows) entrapped in the stapler just before firing

Knowing that the NG tube has been stapled to the sleeve (Figure 2), the 36 Fr gastric calibration tube was reinserted into the sleeve, the staples of the attached area of the NG tube to the sleeve were cut and opened using an ultrasonic scissors creating a rent of around 1 cm which released the proximal NG tube and could be retrogradely pulled out under vision. The gastric calibration tube was kept in situ and the rent on the sleeve was sutured using 2-0 polyglycolic sutures in two layers. Leak test was re-done and ports were closed after inserting an abdominal drain. She had some postoperative reflux which settled with conservative treatment and check endoscopy after six weeks showed no evidence of stricture or ulceration at the site of suturing. 

## DISCUSSION

LSG is a very common operation and is regarded by many as the final surgery required for patients with morbid obesity^1^ ^,^ ^3^. It comes with its own complications and paramount among them is a leak, which is comparatively to other techniques of bariatric surgery, probable in the proximal part of the long staple line^1^ ^,^ ^2^. However, rare complications like NG tube sectioning and stapling to the sleeve though possible is reported only once before this case^4^. In the only previous report the severed NG tube could only be detected in the postoperative period outside the operation room and hence they took the remnant out using an endoscope and closing laparoscopically.

### Why did it happen?

On reanalyzing the video of the surgery, it was very clear that the 4^th^ stapler (blue) had the NG tube in its jaws and was fired on the NG tube while creating the sleeve (Figure 2). But the most important part was to check, how the NG tube reached the stomach during the stapling? 

We have a protocol of inserting a NG tube at the time of induction of anaesthesia to decompress the stomach which is taken out completely after all the ports are inserted and check laparoscopy done. Unfortunately on that day the anaesthetist had withdrawn the NG tube partially and kept it hanging in the oesophagus for a probable later use. When he pushed the gastric calibration tube before firing the staplers, the larger size gastric calibration tube dragged the NG tube into the stomach. Unknowingly we concentrated on the larger gastric tube and fired over the NG tube only to see this unsual complication.

### How to prevent? Is NG tube necessary?

Prevention of such unusual complication is of paramount importance; hence awareness among surgeons that the NG tube can be severed without any pressure by the modern day stapler makes it even more necessary for its careful application during the procedure. The absolute answer to it would be to completely taking out the NG tube before inserting the calibration tube and all leak tests to be done with the calibration tube itself. Further the role and necessity of the NG tube before the procedure to achieve gastric decompression and prevention of leak needs to be evaluated^4^. 

### How to recover from such happening?

These complications, though rare, can happen with any surgical team. As its rare, definite recovery protocols cannot be compared, however without fail, as discussed, prevention is always better proposition than recovery. In our case we think that the in situ gastric calibration tube which we had inserted knowing that the NG tube has been stappled acted as a stent and helped us in maintaining the sleeve, facilitating detaching the NG tube initially or preventing any injury to the nearby otherwise folded gastric mucosa and later during suturing of the created rent preventing narrowing at the site.
